# miR-101 sensitizes A549 NSCLC cell line to CDDP by activating caspase 3-dependent apoptosis

**DOI:** 10.3892/ol.2013.1725

**Published:** 2013-12-03

**Authors:** JIQING YIN, MINGGUO WANG, CUIXIANG JIN, QINGGUO QI

**Affiliations:** 1 Department of General Practice, Shandong University Hospital, Shandong University, Jinan, Shandong 250061, P.R. China; 2 Department of Stomatology, Subsidiary Central Hospital, Shandong University, Jinan, Shandong 250061, P.R. China; 3 Department of Stomatology, Stomatology Hospital of Shandong University, Jinan, Shandong 250061, P.R. China

**Keywords:** miRNA, CDDP, miR-101, chemosensitivity, A549, NSCLC

## Abstract

MicroRNA-101 (miR-101) is evidently downregulated in several types of cancer, including non-small cell lung cancer (NSCLC), and is crucial in sensitizing cells to chemotherapy drugs. The aim of the present study was to investigate the correlation between miR-101 and chemosensitivity in the A549 NSCLC cell line. Here, we used the human A549 cell line for transfection with an miR-101 overexpressing vector and detected the cytotoxic acticity, proliferation and apoptosis of *cis*-diaminedichloroplatinum (CDDP) in A549-miR-101 and A549-mock cells. We demonstrated that overexpression of miR-101 sensitized A549 cells to CDDP, one of the most frequently used agents in curing or controlling NSCLC and enhanced CDDP-induced cell death and caspase 3-dependent apoptosis. In addition, miR-101 facilitated the inhibitory role of CDDP in A549 cell colony formation. Overall, the results of the present study demonstrated that miR-101 sensitizes the A549 NSCLC cell line to CDDP via the activation of caspase 3-dependent apoptosis.

## Introduction

Lung cancer is the most common type of malignant tumor and the leading cause of cancer-related mortality worldwide (1,2). Non-small cell lung cancer (NSCLC) accounts for ~80% of primary lung cancer, and approximately two-thirds of NSCLC patients are diagnosed in advanced stages, which may contribute to high mortality levels. Chemotherapy and radiation therapy are useful treatments for patients with NSCLC. The chemotherapeutic drug, *cis*-diaminedichloroplatinum (CDDP), is one of the most frequently used agents in curing or controlling NSCLC, but NSCLC is insensitive to CDDP-based chemotherapy in a clinical setting, which is a major clinical obstacle for the successful treatment of NSCLC. The underlying mechanism has not been determined (3,4).

MicroRNAs (miRNAs) are a class of small, single-stranded, non-coding RNAs of 19–25 nt that are involved in the regulation of cellular development, proliferation, differentiation, apoptosis and metabolism (5–8). miRNAs downregulate target mRNAs by interacting with their 3′-untranslated regions through sequence-specific base-pairing (9). Therefore, miRNAs are pivotal in regulating gene expression and it is estimated that miRNAs regulate ~30% of all protein-coding genes (10). In previous years, accumulating evidence has implicated that dysregulation of miRNAs is associated with the initiation and progression of cancer (11,12). miRNA-101 (miR-101) belongs to a family of miRNAs that are involved in a series of cellular activities, such as cell proliferation, invasion and angiogenesis (13,14). Previously, it has been found that miR-101 is expressed in several types of cancer, including liver, prostate and breast, and emerging evidence indicates that miRNAs may act as cancer suppressors (15–17). In addition, it has been reported that miR-101 induces apoptosis and suppresses tumorigenicity *in vitro* and *in vivo,* and inhibits the migration and invasion of gastric cancer cells (15). Notably, studies have shown that miR-101 is evidently downregulated in NSCLC, and it inhibits cell proliferation and invasion. Moreover, miR-101 enhances paclitaxel-induced apoptosis in NSCLC cells by directly repressing the enhancer of zeste homolog 2 expression (18,19). In addition, the role of miR-101 in chemosensitivity has been previously identified. Batchu *et al* reported that miR-101 enhances the chemosensitivity of pancreatic ductal adenocarcinoma (PDAC) cells by inhibition of mammalian target of rapamycin (mTOR) signaling via proline-rich Akt substrate 40 (PRAS40) (20). Xu *et al* reported that miR-101 enhanced apoptosis induced by CDDP in the HepG2 cell line by inhibiting autophagy (21). However, limited knowledge is available concerning whether miR-101 expression affects the chemosensitivity of NSCLC, and the underlying molecular mechanism remains unclear.

In the present study, we provide further evidence that miR-101 enhances the chemosensitivity of A549 NSCLC cells to CDDP. Furthermore, miR-101 was shown to significantly promote CDDP-induced apoptosis and suppress the colony formation by activating the caspase 3-dependent apoptosis pathway.

## Materials and methods

### Cell culture and transfection

The A549 cell line was derived from adenocarcinomas of the lung obtained from the China Center for Type Culture Collection (Wuhan, China). The lung cancer cells (A549) were maintained in RPMI-1640 medium (Gibco-BRL, Carlsbad, CA, USA) with 10% fetal bovine serum and antibiotics (100 U/ml penicillin and 100 μg/ml streptomycin) within a humidified atmosphere containing 5% CO_2_ at 37°C. Transfection of A549 cells with plasmids, pre-miR-101 or scrambled pre-miR control (GenePharma, Shanghai, China), was performed using Lipofectamine 2000 according to the manufacturer's instructions (Invitrogen Life Technologies, Carlsbad, CA, USA). Untransfected A549 cells were employed as a negative control.

### Total RNA extraction and real-time polymerase chain reaction (qPCR)

Total RNA was extracted from cells using a modified TRIzol one-step extraction method (Invitrogen Life Technologies). Stem-loop reverse transcription for mature miR-101 and U6 primers was performed as previously described. U6 RNA was used as an miRNA internal control. The primers used for stem-loop reverse-transcription PCR for miR-101 were purchased from Guangzhou RiboBio Co., Ltd. (Guangzhou, China). Each sample was conducted in triplicate and the results were calculated using the 2^−ΔΔCt^ method.

### 3-(4,5-dimethylthiazol-2yl)-2,5-diphenyl tetrazolium bromide (MTT) assay

The effect of miR-101 on cell growth was measured by MTT assay. A549 cells that had been mock-transfected or transfected with pre-miR-101 were seeded respectively into 96-well plates at a density of 5×10^3^ cells/well and allowed to grow overnight. Cells were then treated with various concentrations of CDDP (QiLu Pharmaceutical Co., Ltd., Jinan, China). Following 24 h of CDDP treatment, 20 μl MTT (5 mg/ml; Sigma-Aldrich, St Louis, MO, USA) was added to the cells, which were incubated in the dark for 4 h. Following the removal of the cell supernatants, formazan crystals were dissolved in 150 μl dimethylsulfoxide. The viability of treated cells was calculated from the average OD570 values compared with those of the untreated cells with an enzyme-linked immunosorbent assay reader (Infinite M200; Tecan Group Ltd., Männedorf, Switzerland). Each group was run in triplicate wells.

### Colony formation assay

A549 cells were transfected and treated with CDDP. Following 24 h of treatment, cells were reseeded into six-well plates with a density of 500 cells per well for 2–3 weeks. The medium was discarded and each well was carefully washed twice with phosphate-buffered saline (PBS). The colonies were fixed in methanol for 20 min and then stained with Giemsa staining solution. The number of colonies with ≥50 cells were counted and colony forming efficiency was calculated using the following formula: Percentage of colonies (%)= number of colonies formed/number of cells inoculated ×100. Experiments were repeated three times.

### Cell death analysis

Cell death was assessed by flow cytometry using propidium iodide (PI). A549 cells were transfected and treated with CDDP as described above. The cells from various treated groups were collected and washed twice with cold PBS. The cells were then resuspended in binding buffer at a concentration of 1×10^6^ cells/ml. In total, 100 μl cell suspension (1×10^5^ cells) was transferred to a 5-ml culture tube. Following this, 10 μl PI was added to the cell suspension and incubated for 15 min at room temperature in the dark. The stained cells were then diluted to 500 μl with binding buffer and analyzed with a FACSCalibur flow cytometer (Beckman Coulter, Miami, FL, USA). Triplicate assays were performed.

### Apoptosis detection by terminal deoxynucleotidyl transferase-mediated dUTP nick end labeling (TUNEL) staining

To evaluate the degree of CDDP-induced apoptosis, TUNEL assays were performed. A549 cells were incubated in six-well plates and transfected as previously described. Following 24 h of transfection and then 24 h with or without CDDP stimuli exposure, the cells in each well were washed twice with cold PBS and fixed with 4% (v/v) formaldehyde in PBS for 10 min at room temperature. TUNEL was performed using the In Situ Cell Death Detection Kit, Fluorescein (Roche Diagnostics, Mannheim, Germany) according to the manufacturer's instructions. Randomly selected microscopic fields (n=8) were counted under a fluorescent microscope (Leica, Wetzlar, Germany) and the ratio of TUNEL-positive cells to the total number of cells was calculated in three independent experiments using ImageJ software (National Institutes of Health, Bethesda, MD, USA).

### Western blot analysis

A549 cells from various treated groups were washed twice in cold PBS and lysed for 20 min using RIPA buffer (Thermo Fisher Scientific, Waltham, MA, USA), with freshly added protease inhibitor and phosphatase inhibitor (Roche Diagnostics, Indianapolis, IN, USA). Protein concentration in the cell lysate was determined using the BCA assay (Pierce Biotechnology, Inc., Rockford, IL, USA). In total, 50 μg of protein was separated on SDS-PAGE and then transferred onto PVDF membranes (Millipore, Billerica, MA, USA). Next, the membranes were blocked with 2% BSA in TBST containing 0.1% Tween-20 for 1 h at room temperature. Membranes were then incubated overnight at 4°C with antibodies against caspase 3, (1:1,000; Cell Signaling Technology, Inc., Beverly, MA, USA) and β-actin (1:2,000; Santa Cruz Biotechnology, Inc., Santa Cruz, CA, USA). After washing, the membranes were incubated with horseradish peroxidase-conjugated secondary antibody (1:2,000) followed by ECL detection (Amersham Pharmacia Biotech, Amersham, UK).

### Statistical analysis

All statistical analyses were conducted using SPSS 11.5 software (SPSS, Inc., Chicago, IL, USA). All numerical data were generated from three independent experiments and are presented as means ± standard error of the mean. Differences between groups were examined using one-way analysis of variance or Student's t-test, as appropriate. P<0.05 was considered to indicate a statistically significant difference.

## Results

### Overexpression of miR-101 correlates with cytotoxic activity of CDDP in A549 cells

**Figure 1:**
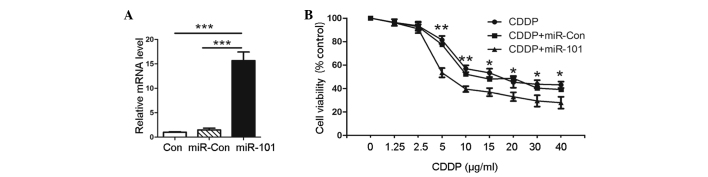
Effect of the overexpression of miR-101 on the cytotoxic activity of CDDP in A549 cells. (A) Verification of miR-101 transfection. The expression levels of miR-101 were confirmed by real-time polymerase chain reaction. miR-101-transfected cells showed higher miR-101 expression than the Con (P<0.01) and miR-Con (P<0.01) groups. (B) Cell viability was detected by 3-(4,5-dimethylthiazol-2yl)-2,5-diphenyl tetrazolium bromide assay in pre-miR-101 transfected cells and Con and miR-Con groups following treatment with CDDP for 24 h. Data are presented as the mean ± SEM and experiments were performed in triplicate. ^*^P<0.05 and ^**^P<0.01, between CDDP + miR-101 and CDDP + miR-Con groups. ^***^P<0.001. miR-101, microRNA-101; Con, untransfected negative control; miR-Con, empty vector-transfected; CDDP, *cis*-diaminedichloroplatinum.

In order to explore the role of miR-101 in A549 cells, transfection with plasmids, pre-miR-101 or scrambled pre-miR control, was performed. The expression levels of miR-101 were confirmed by qPCR, as shown in Fig. 1A. Pre-miR-101 (miR-101)-transfected cells showed a higher miR-101 expression than the untransfected negative control (Con) and empty vector-transfected (miR-Con) groups. To evaluate the effect of miR-101 on the cytotoxic activity of CDDP in A549 cells, MTT assay was performed on the pre-miR-101 transfected cells and the Con and miR-Con groups combined with various concentrations of CDDP. The results showed that the viability of A549 cells with miR-101 overexpression was significantly decreased compared with that of the miR-Con or Con groups at the same concentration of CDDP (Fig. 1B). This indicated that miR-101 sensitizes A549 cells to CDDP compared with the Con group. This observation suggested that the overexpression of miR-101 facilitates the cytotoxic activity of CDDP in A549 cells.

### Overexpression of miR-101 in A549 cells increases CDDP-induced cell death

**Figure 2:**
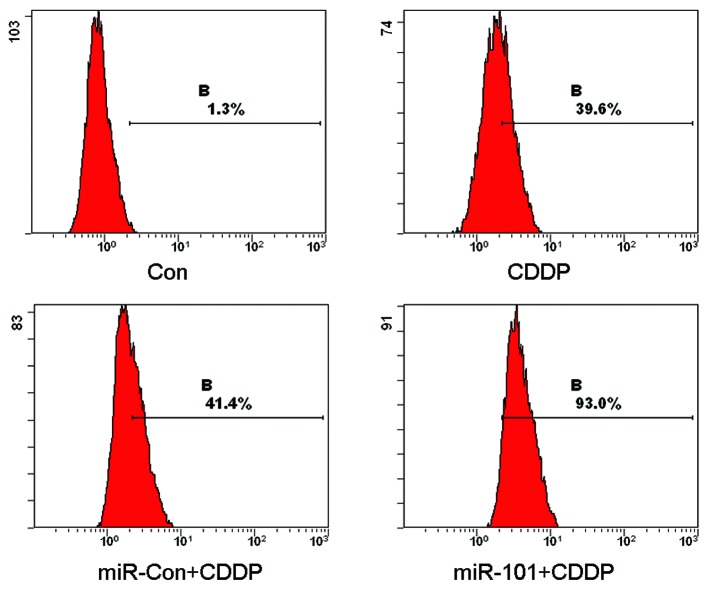
Overexpression of miR-101 in A549 cells increases CDDP-induced cell death. CDDP-induced cell death was assessed by flow cytometry. Following treatment with CDDP for 24 h, miR-101-transfected cells showed a higher ratio of cell death (93.0%), whereas negative controls exhibited a lower cell death ratio: Con (1.3%), CDDP (39.6%) and CDDP plus miR-Con (41.4%). Con, untransfected negative control; miR-Con, empty vector transfected; CDDP, *cis*-diaminedichloroplatinum.

In order to explore the manner in which the modulation of miR-101 in the A549 cell line affected CDDP-induced cell death, pre-miR-101 transfected cells and the Con and miR-Con groups were treated with CDDP and analyzed by flow cytometry. The results of the flow cytometry revealed that the mean rates of cell death were 1.3, 39.6, 41.4 and 93.0% for the Con, CDDP, CDDP plus miR-Con and CDDP plus miR-101 groups, respectively (Fig. 2). These results clearly indicated that the overexpression of miR-101 enhances CDDP-induced cell death in A549 cells.

### Overexpression of miR-101 promotes CDDP-induced caspase 3-dependent apoptosis

**Figure 3:**
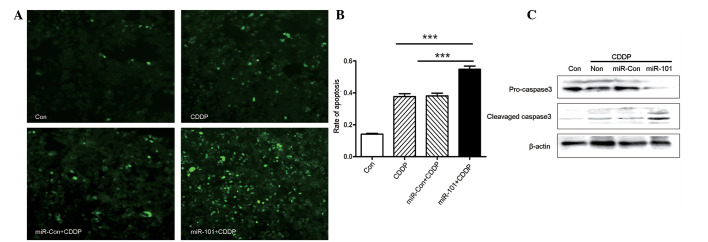
Apoptotic analysis. (A) Apoptosis of A549 cells from various treated groups was detected by terminal deoxynucleotidyl transferase-mediated dUTP nick end labeling staining. Results showed increased positive signals in A549 cells overexpressing miR-101 compared with those in the vector control cells (P<0.01). Data shown are representative of three independent experiments. (B) Number of apoptotic cells, indicated by the bright green fluorescence, and total cell number were quantified using the ImageJ programe, and the rate of apoptosis was subsequently calculated. The results are presented as means ± SD. (C) Western blot analysis results showed increased cleaved caspase 3 expression in A549 cells overexpressing miR-101 compared with that in the vector control cells. ^***^P<0.001, vs. control and CDDP groups. CDDP, *cis*-diaminedichloroplatinum; Con, untransfected negative control without CDDP; Non, untransfected control with CDDP; miR-Con, empty vector-transfected.

To explore whether miR-101 expression may alter CDDP-induced apoptosis, apoptosis was measured by TUNEL staining. As shown in Fig. 3A and B, miR-101 overexpression increased the number of apoptotic cells following treatment with CDDP. In addition, western blot analysis demonstrated that miR-101 was involved in caspase 3-dependent apoptosis, as shown in Fig. 3C. CDDP led to an increased level of cleaved caspase 3 in A549-miR-101 cells, to a greater extent than that in A549 or A549-miR-Con cells. Overall, miR-101 overexpression enhanced chemosensitivity to CDDP in the A549 NSCLC cancer cell line via caspase 3-dependent apoptosis.

### Overexpression of miR-101 inhibits A549 cell colony formation

**Figure 4:**
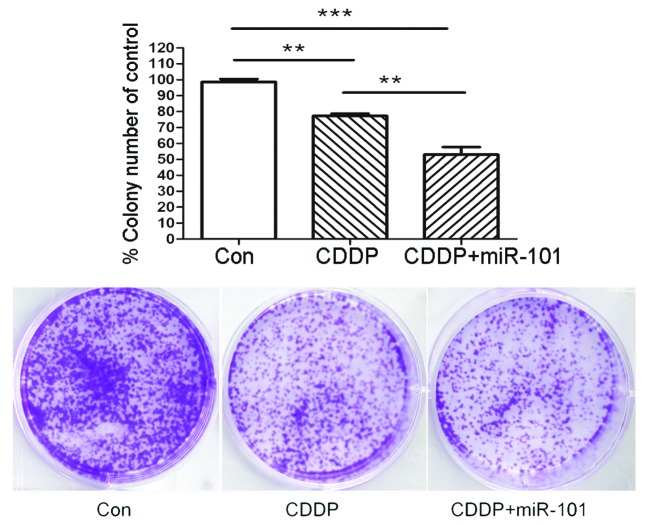
Overexpression of miR-101 inhibits A549 cell colony formation. Colonies were allowed to grow for 14–21 days and positive colony formation (>50 cells/colony) was counted. The results indicated that overexpression of miR-101 significantly diminished the colony formation rates of A549 cells compared with the mock group. Data are presented as the mean ±SD and experiments were performed in triplicate. ^**^P<0.01 and ^***^P<0.001. miR-101, microRNA-101; Con, untransfected negative control; CDDP, *cis*-diaminedichloroplatinum.

To confirm the long-term survival rate of cells, a colony assay was performed in the A549 cell line with various treatments as described previously. As shown in Fig. 4, overexpression of miR-101 in A549 cells caused a significant reduction in the number and diameter of the colonies at day 14 compared with those of the Con and miR-Con groups.

## Discussion

Although chemotherapeutic agents, including CDDP, are widely used in the treatment of lung cancer, their efficacy is often limited by the existence or development of chemoresistance. Factors that enhance the sensitivity of NSCLC cells to chemotherapeutic drugs may highlight predictive biomarkers or targets for therapy. miR-101 is an miRNA, which has been previously demonstrated to regulate a variety of biological processes by modulating the expression of several target genes (22,23). A growing number of studies have implicated that miR-101 may function as a tumor suppressor gene to inhibit the expression of tumor-promoting genes. In addition, miR-101 has been demonstrated to be downregulated in several types of human cancer, including lung cancer. Emerging evidence suggests that miR-101 induces apoptosis, suppresses tumorigenicity, inhibits migration and invasion, and is crucial in promoting chemosensitivity (15,17,24–26). The present study has demonstrated for the first time that miR-101 sensitizes the A549 NSCLC cell line to CDDP.

To date, the mechanism by which miR-101 enhances chemosensitivity remains unclear. Previously, Batchu *et al* reported that miR-101 enhances the chemosensitivity of PDAC cells by inhibition of mTOR signaling via PRAS40 (20). Xu *et al* reported that miR-101 enhanced apoptosis induced by CDDP in the HepG2 cell line by inhibiting autophagy (21). In order to explore whether miR-101 expression affects the chemosensitivity of lung tumors, the human A549 cell line was used for transfection with the miR-101 overexpression vector. The cytotoxic activity, proliferation and apoptosis of CDDP were then examined in A549-miR-101 and A549-mock cells. MTT assays demonstrated that A549-miR-101 and A549-mock cells were differentially sensitive to CDDP. It was also found that miR-101 overexpression led to cell death, caspase 3-dependent apoptosis to CDDP and inhibited cell colony formation. The evidence that high miR-101 levels result in drug sensitivity indicated that miR-101 may regulate the sensitivity of chemotherapeutic drugs in NSCLC. Overall, the results of the current study show that miR-101 overexpression effectively promotes CDDP-induced apoptosis and inhibits colony formation in the A549 NSCLC cell line.

In conclusion, the present study demonstrated for the first time that miR-101 functions as an inducer of chemotherapeutic sensitivity in NSCLC by activating the caspase 3-dependent apoptosis pathway. These results established that miR-101 transfer in combination with CDDP therapy may be a target to reverse chemotherapeutic resistance. Further investigations with regard to the miR-101 regulation of chemosensitivity are likely to provide insights into the mechanistic details of this regulatory network.

## Abbreviations

miR-101: microRNA-101
NSCLC: non-small cell lung cancer
CDDP: *cis*-diaminedichloroplatinum
PDAC: pancreatic ductal adenocarcinoma
mTOR: mammalian target of rapamycin
PRAS40: proline-rich Akt substrate 40
